# Culturable Fungal Community of *Pterocladiella capillacea* in Keelung, Taiwan: Effects of Surface Sterilization Method and Isolation Medium

**DOI:** 10.3390/jof7080651

**Published:** 2021-08-11

**Authors:** Hyo-Jung Cha, Michael W. L. Chiang, Sheng-Yu Guo, Showe-Mei Lin, Ka-Lai Pang

**Affiliations:** 1 Centre of Excellence for the Oceans and Institute of Marine Biology, National Taiwan Ocean University, 2 Pei-Ning Road, Keelung 202301, Taiwan; hyojung.a@gmail.com (H.-J.C.); alexsyguo@gmail.com (S.-Y.G.); linsm@ntou.edu.tw (S.-M.L.); 2 Department of Chemistry, City University of Hong Kong, 83 Tat Chee Avenue, Kowloon Tong, Hong Kong; bhchiang@cityu.edu.hk

**Keywords:** culture dependent, fungal community, marine fungi, rhodophyta, symbiosis

## Abstract

Fungi associated with macroalgae are less known when compared with those on wood in the marine environment. In this study, we assessed the diversity of fungi associated with the red alga *Pterocladiella capillacea* at Chao-Jin Park, Keelung, Taiwan. Algal segments of healthy and dead thalli were washed/sterilized with different solutions (sterile artificial seawater, 70% ethanol, and 4% sodium hypochlorite), plated on three different media (glucose-yeast extract-peptone seawater agar (GYPS), potato dextrose seawater agar (PDAS), and artificial seawater agar (SA)), and isolated as pure cultures. Identification was mainly based on BLAST search analysis of the internal transcribed spacers of rDNA (ITS). The highest isolation frequency (no. of segment with fungi/total no. of segment × 100) was in dead thalli (61.23%), thalli washed with seawater (88.38%), and thalli plated on GYPS (62.10%). A total of 3187 isolates were cultured, representing 129 taxa (in 67 genera); the higher species richness was isolated from healthy thalli (119 species), thalli washed with seawater (111 species), and on GYPS (112 species). Ascomycota (Eurotiales, Hypocreales, Capnodiales, Pleosporales, Xylariales) dominated the fungal community in *P. capillacea* with many basidiomycetous yeasts and few Mucoromycota. *Aspergillus*, *Cladosporium*, *Penicillium* (Ascomycota), and *Rhodosporidium* (Basidiomycota) were the dominant genera associated with the alga. The surface washing/sterilization schemes of algal thalli affected fungal diversity, but the isolation media used did not. While these genera are known producers of antimicrobial secondary metabolites, they might form a mutualistic relationship with *P. capillacea* by exchanging nutrients from photosynthesis for protection from microbial diseases.

## 1. Introduction

Early studies of marine fungi mainly focused on those associated with macroalgae [1,2]. Macroalgae are particularly abundant in coastal environments and a good substrate for growth of microorganisms including marine fungi [3]. Fungi on macroalgae are mainly saprobic, although many species are probably symbiotic, including pathogenic/parasitic associations [4,5]. Saprobic marine fungi of macroalgae form fruiting bodies on the substrate and produce a unique morphology, such as species of the genera *Spathulospora*, *Chadefaudia*, *Haloguignardia*, *Retrostium*, *Histopidicarpomyces*, and *Pontogenia* [4]. Concerning pathogenic species of macroalgae, *Mycaureola dilseae* is a marine basidiomycete parasitizing the marine alga *Dilsea carnosa*, forming circular necrotic lesions [6]. *Lautitia danica* occurs on the red alga *Chondrus crispus* throughout the whole year, forming ascomata on the fronds [7]. Many filamentous fungi can also asymptomatically colonize the algal inner tissues without causing any apparent damage or disease [8,9]. Protrusions of the cell wall of the ascomycete *Verrucaria tavaresiae* penetrate walls of the brown alga *Petroderma maculiforme*, possibly forming a symbiotic relationship [10]. *Mycophycias ascophylli* and *Sigmoidea marina* were found to be mutually associated with their algal host and grow within or at the surface of the algal tissue [11,12].

In recent years, fungi have been isolated from surface-sterilized and seawater-washed thalli (pieces) of macroalgae for their production of interesting antimicrobial and/or cytotoxic metabolites [13,14]. Loque et al. [15] and Godinho et al. [16] studied the fungi associated with macroalgae at Antarctic regions, and the common fungi were *Geomyces*, *Penicillium,* and *Metschnikowia*. *Meyerozyma guilliermondii* was found to be a common yeast [17]. On the red algal genus *Kappaphycus* (*K*. *alvarezii*, *K*. *striatum*) in the Philippines, the common fungi were *Fusarium* sp., *Curvularia intermedia*, *Cladosporium* sp., and *Phoma* sp. [18]. Abdel-Gawad et al. [19] isolated fungi from 20 macroalgae consisting of 8 red algae, 8 brown algae, and 4 green algae collected along the coast of the Red Sea in Egypt, the dominant genera were *Penicillium*, *Alternaria*, *Cladosporium*, *Aspergillus,* and *Acremonium*. The above studies used seawater to wash the algae prior to inoculation and resulted numerous typical terrestrial genera, many of which can grow better in the presence of sea salts [18]. However, Zuccaro et al. [20], on the study of fungi associated with *Fucus* spp., suggested that *Emericellopsis* spp. and *Acremonium* spp. on *Fucus* were distinct from their terrestrial counterparts physiologically and phylogenetically.

Many macroalgae have been examined for their endophytic association with fungi, where the algal surface was sterilized prior to isolation. In the Shetland Islands, UK and Bay of Fundy, Canada, *Aspergillus* and *Penicillium* species were common on macroalgae [21,22]. In the same studies, *Mycosphaerella ascophylli* was not isolated from the brown alga *Ascophyllum nodosum,* although this is a common marine ascomycete reported to associate with this alga endophytically [7]. *Aspergillus* spp. were also found to be dominant endophytically in 24 species of red, green, and brown algae collected at Tamilnadu, India, together with an unidentified yeast species [23]. Asexual Ascomycota was abundant on macroalgae, either occurring epiphytically or endophytically [19,23].

Kohlmeyer and Volkmann-Kohlmeyer [24] listed 75 species of marine fungi associated with algae either parasitically or symbiotically. This estimate is restricted to those fungi forming visible fruiting bodies on the algae. Jones et al. [5] provided a more updated figure of 112 taxa, including those parasitic or saprophytic on algae and those forming lichenized relationships with algal hosts. The Ascomycota is the dominant phylum of fungi cultured from microalgae [12,16,21,22,25], such as *Acremonium*, *Alternaria*, *Arthrinium*, *Aspergillus*, *Cladosporium*, *Emericellopsis*, *Fusarium*, *Penicillium*, *Phoma*, *Pontogenia*, *Retrosium*, *Spathuospora*, *Sigmoidea*, and *Trichoderma* [7,12,20,24,26,27,28].

Roughly, there are 7000 described species of the Rhodophyta, and only a few have been examined for their associated fungi [29]: *Ballia*, *Ceramium*, *Chondrus*, *Dilsea*, *Gelidiella*, *Gracilaria*, *Grateloupia*, *Halymenia*, *Hypnea*, *Porphyra*, and *Portieria* [6,7,23,27]. *Pterocladiella capillacea* is a red alga growing throughout the year along the rocky shore of northern Taiwan. In this study, we examined the culturable fungal community associated with dead and healthy thalli of *P. capillacea* washed with sterile seawater, 70% ethanol, or 4% sodium hypochlorite (NaOCl) before inoculation and plated on glucose-yeast extract-peptone seawater agar (GYPS), potato dextrose seawater agar (PDAS), and artificial seawater agar (SA) collected bimonthly over a 1-year period. Sterile seawater, ethanol, and sodium hypochlorite were the most common chemical agents, either individually or in combination, to treat samples of macroalgae before they were inoculated onto isolation media, but concentrations of the chemical agents and duration of treatment differed between studies [15,21,22,23,30]. Sodium hypochlorite was found to be an effective surface sterilizer of samples and has been used in studies targeting endophytic fungal assemblages [31]. However, a combination of 70% ethanol for 5 s and 4% sodium hypochlorite for 60 s and a long surface sterilization time of 60 s caused visible damage of algal tissue [23]. In this study, the use of seawater, 70% ethanol (30 s), and 4% sodium hypochlorite (10 s) to wash/surface sterilize algal samples enabled detection of both epiphytic and endophytic fungi of *P. capillacea*. Potato dextrose agar, malt extract agar, and glucose-yeast extract-peptone agar have been some of the most commonly used nutritious media for isolation of fungi from macroalgae [15,18,19,21,22,32,33,34,35], although other media have also been used, such as marine agar [16] and cornmeal agar [36]. To broaden the isolation of taxonomically diverse fungi, three media, i.e., potato dextrose agar, glucose-yeast extract-peptone agar, and seawater agar, were used in this study. Fungi were isolated as axenic cultures and identified based on a BLASTn analysis of the internal transcribed spacers of rDNA (ITS), as well as 18S and/or 28S rDNA when the identity provided by ITS alone was ambiguous.

## 2. Materials and Methods

### 2.1. Collection of Samples

**Figure 1:**
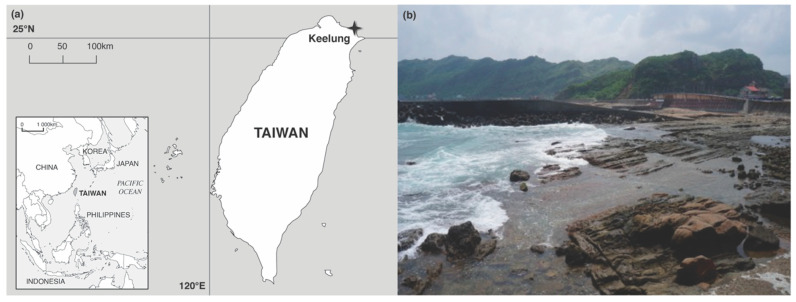
Sampling site: (**a**) Sampling location (star) of *Pterocladiella capillacea* along Chao-Jin Park, Keelung, northern Taiwan. (**b**) Photograph of the sampling site.

Algal thalli of *Pterocladiella capillacea* were collected bimonthly between May 2013 to May 2014 at Chao-Jin Park, located at the east side of the Badouzi Peninsula facing Wanghaixiang Bay (25°08′31.9″ N, 121°48′08.7″ E), Keelung, northern Taiwan (Figure 1). Healthy (red color) and dead or decaying (white color) thalli were collected along with seawater, placed in sterilized polythene bags, and maintained at 4 °C during transportation to the laboratory for immediate isolation.

### 2.2. Surface Washing/Sterilization of Pterocladiella Capillacea

Fungi isolation was conducted based on the conventional methodology (Figure S1). The collected healthy and dead thalli were cut into segments of approximately 0.5–1.0 cm^2^. The segments were subjected to three different surface washing/sterilization procedures: (1) washed in sterile artificial seawater for 30 s, (2), immersed in 70% ethanol for 30 s, and (3) immersed in 4% sodium hypochlorite for 10 s. All samples were then further rinsed two times with sterile artificial seawater for 30 s each.

### 2.3. Fungal Isolation

After washing/sterilization, randomly selected segments were plated onto three different media on Petri dishes using a flame sterilized forceps: seawater agar (SA; 30 g/L artificial sea salt, 15 g/L agar (Bioshop, Burlington, ON, Canada)), glucose-yeast extract-peptone seawater agar (GYPS; 4 g/L glucose (Bioshop, Burlington, ON, Canada), 4 g/L yeast extract (Oxoid, United Kingdom), 4 g/L peptone (Oxoid, United Kingdom), 30 g/L artificial sea salt, 15 g/L agar (Bioshop, Burlington, Ont, Canada)), and potato dextrose seawater agar (PDAS; 39 g/L potato dextrose agar (Becton, Dickinson and Company, Sparks, MD, USA), 30 g/L artificial sea salt) supplemented with 0.5 g/L each of streptomycin sulphate and Penicillin G sodium salt (Bioshop, Burlington, ON, Canada). The inoculated plates were incubated at 25 °C and observed periodically for up to 1 month under a stereomicroscope (Olympus, Tokyo). Fungi with different fungal mycelial morphologies were cut out and subcultured onto cornmeal seawater agar (CMAS; 17 g/L corn meal agar (Himedia, India), 30 g/L artificial sea salt) plates as pure cultures. All cultures are kept at the Institute of Marine Biology, National Taiwan Ocean University (NTOU).

### 2.4. Fungal Identification

The isolates on CMAS were categorized into morphotypes based on their colony morphology. Total genomic DNA of each morphotype was extracted using a DNeasy^®^ Plant Mini Kit according to the manufacturer’s instructions (Cat. No. 69104, Qiagen, Germany). Using the genomic DNA as the template, the internal transcribed spacer (ITS) regions spanning from the end of the 18S rDNA to the beginning of the 28S rDNA for each morphotype were amplified using the primer pairs ITS5/ITS4 [37] or ITS1-F_KYO1/ITS4_KYO3 [38]. In some cases, the 18S and 28S rDNA were amplified using NS1/NS6 [37] and LR0R/LR6 [39], respectively. Thermal cycling conditions for PCR amplification were set as follows: 95 °C for 2 min, and 35 cycles of 95 °C for 60 s, 54 °C for 60 s, 72 °C for 90 s, and a final extension at 72 °C for 10 min. The presence or absence of a DNA band corresponding to the ITS size (approximately 550 base pairs) was verified through a gel electrophoresis (run time 25 min; 120 V) in an 1% agarose gel (Tris-Acetate EDTA (TAE) buffer, 5 µL SYBR^®^Safe (Invitrogen, United States)). Positive PCR products were sent to Genomics BioSci and Tech (New Taipei City, Taiwan) for sequencing. The sequences obtained were checked for ambiguity, assembled, and submitted to the National Center for Biotechnology Information (NCBI) for a nucleotide BLAST search.

### 2.5. Statistics

Isolation frequency (IF) is calculated as the number of algal segments with fungal growth over the total number of algal segments examined and expressed as a percentage [40].

$$\mathrm{IF}\;\left(\%\right)=\frac{\mathrm{Number}\;\mathrm{of}\;\mathrm{algal}\;\mathrm{segments}\;\mathrm{showing}\;\mathrm{fungal}\;\mathrm{growth}\;}{\mathrm{Total}\;\mathrm{number}\;\mathrm{of}\;\mathrm{algal}\;\mathrm{segments}}\times 100$$

Percentage occurrence (%) is the number of isolates of each fungal taxon divided by the total number of isolates of all taxa at each sampling time.

$$\mathrm{Percentage}\;\mathrm{occurrence}\;\left(\%\right)=\frac{\mathrm{Number}\;\mathrm{of}\;\mathrm{isolates}\;\mathrm{for}\;a\;\mathrm{particular}\;\mathrm{fungus}\;}{\mathrm{Total}\;\mathrm{number}\;\mathrm{of}\;\mathrm{fungal}\;\mathrm{isolates}}\times 100$$

Diversity of the species cluster was analyzed by the Shannon–Wiener diversity index (*H′*) [41]; species richness was measured by the Margalef index (*d*) [42]; Pielou’s evenness index (*J*′) [43] and Simpson’s dominance index (*D*) [44] were calculated to determine the heterogeneity of fungal diversity. For comparison of alpha diversity between different isolation conditions, rarefaction analysis was firstly done to check species richness, and the rarefaction and extrapolation curves were constructed based on fungal abundance in the R iNEXT package [45]. Comparisons of the estimated and extrapolated species richness in plots were calculated based on 50 bootstrap replicates at a 95% confidence interval. The significance of the difference between the effects of media, surface washing/sterilization methods, and healthy/dead algal thalli on fungal diversity was tested using the non-parametric Kruskal–Wallis tests (<0.05) and pairwise Wilcoxon test (<0.05). Krona (v.2.6) [46] was adopted to visualize the taxonomic composition of fungi (species richness and percentage occurrence) through the Krona pie chart. The construction of Venn diagrams was performed using a Venn diagram viewer [47].

## 3. Results

### 3.1. Culturable Fungal Diversity of Pterocladiella Capillacea

**Figure 2:**
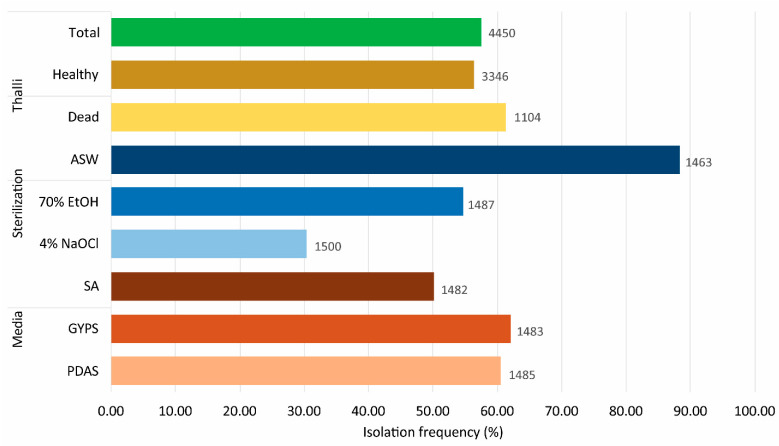
Isolation frequency (%) of fungi from healthy and dead algal fragments after three surface washing/sterilization methods (ASW—artificial seawater, 70% EtOH—70% ethanol, and 4% NaOCl—4% sodium hypochlorite) on different media (SA—seawater agar, GYPS—glucose-yeast extract-peptone seawater agar, and PDAS—potato dextrose seawater agar). The number shown at the bars represents the total number of algal fragments examined under that treatment (see also Table S1).

A total of 4450 algal fragments (1104 dead, 3346 healthy) of *P. capillacea* were inoculated, out of which at least one morphotype grew out from 2562 pieces, giving an isolation frequency of 57.57% (Figure 2, Table S1).

Altogether, 3187 fungal isolates (906 from dead, 2281 from healthy) were cultured. These 3187 isolates were categorized into 262 morphotypes based on colony coloration, pigment formation, and mycelial growth patterns on CMAS. ITS sequence analysis referred these 262 fungal morphotypes to a total of 129 species in 67 genera, while 9 morphotypes were not identified to the genus level and referred to a family/order name (Table S1). The % sequence coverage and % sequence similarity of ITS in the BLAST search were above 95%, except for three species. The highest Shannon–Wiener diversity index (*H’*) was observed from healthy thalli (4.0335), thalli sterilized with 4% sodium hypochlorite (4.0923), and thalli plated on GYPS (4.0107) (Table 1). The Margalef species richness (*d*) was also the highest in healthy thalli (15.2605) and thalli plated on GYPS (15.6356), followed by thalli sterilized with 70% ethanol (15.0577) (Table 1). However, thalli sterilized with 4% sodium hypochlorite (0.9072) had the highest evenness index (*J’*) over those sterilized with 70% ethanol (0.8204).

**Figure 3:**
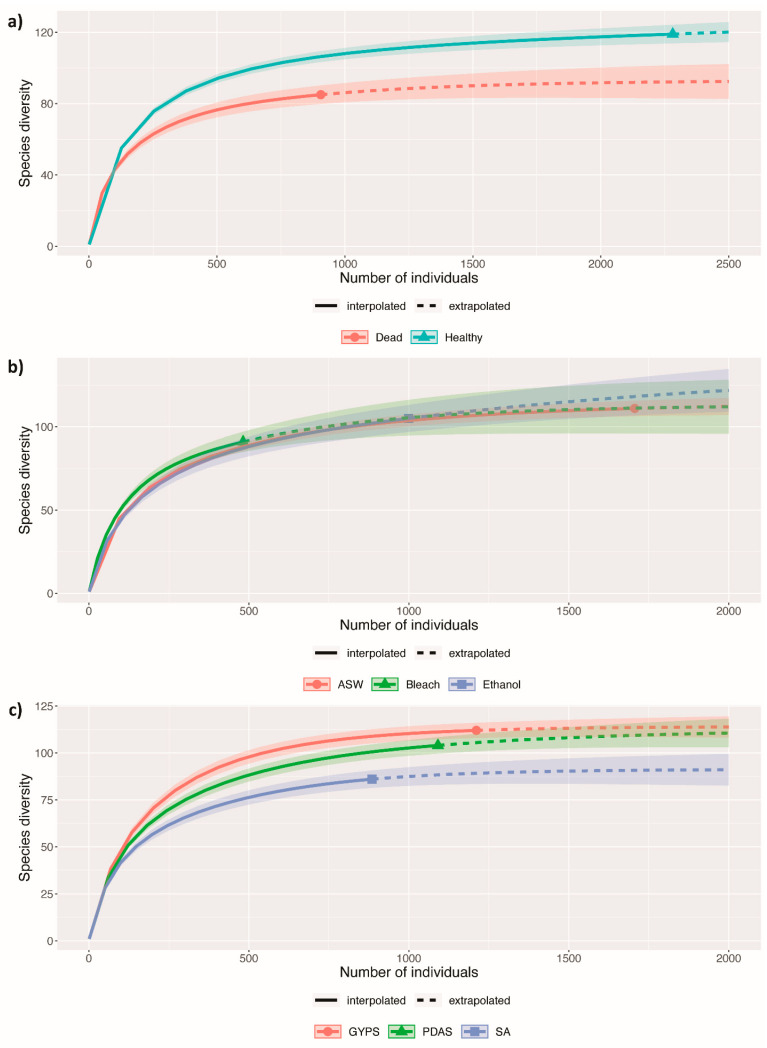
Sample-size-based rarefaction and extrapolation curves of fungal diversity from *Pterocladiella capillacea* for the Hill number (*q* = 0) for each of the different isolation conditions as following (**a**) healthy and dead thalli; (**b**) thalli surface sterilized/washed by artificial seawater (ASW), 70% ethanol (Ethanol), and 4% sodium hypochlorite (Bleach); and (**c**) thalli plated on seawater agar (SA), glucose-yeast extract-peptone seawater agar (GYPS), and potato dextrose seawater agar (PDAS). The 95% confidence intervals were obtained by a bootstrap method based on 50 replicates.

The rarefaction curves calculated from species diversity data shown in Figure 3 revealed species saturation for all treatments (healthy and dead thalli, surface washing/sterilization methods, isolation media)

**Figure 4:**
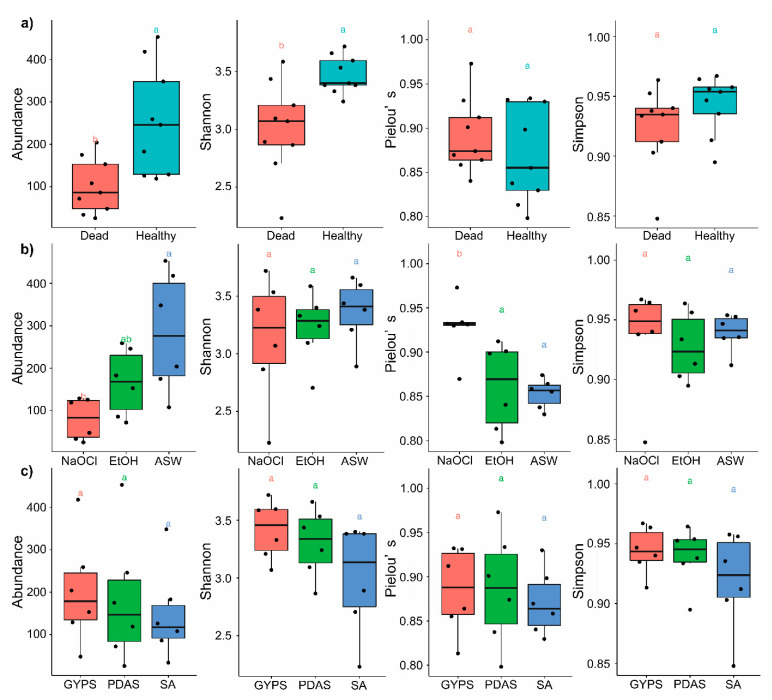
Box plot of alpha diversity indices Shannon, Pielou’s, Simpson, and the relative abundance derived from different conditions of *Pterocladiella capillacea*. The Wilcoxon test was used to determine the significance level between the different groups, while the Kruskal–Wallis test was used to calculate the significance level among the following: (**a**) healthy and dead thalli; (**b**) thalli surface sterilized/washed by artificial seawater (ASW), 70% ethanol (EtOH), and 4% sodium hypochlorite (NaOCl); and (**c**) thalli plated on seawater agar (SA), glucose-yeast extract-peptone seawater agar (GYPS), and potato dextrose seawater agar (PDAS). a, b = same letters indicate no significant differences at *p* < 0.05.

The Kruskal–Wallis analysis showed a statistically significant (*p* < 0.05, Bonferroni corrected) relationship on fungal diversity between healthy and dead thalli from the abundance (*p* = 0.0055) and Shannon diversity (*p* = 0.0056) index, as well as different surface washing/sterilization methods using artificial seawater and 4% sodium hypochlorite from the abundance (*p* = 0.0066) using 4% sodium hypochlorite and artificial seawater (*p* = 0.0063), and using 4% sodium hypochlorite and 70% ethanol (*p* = 0.013) from Pielou’s evenness. However, there was no significant difference on fungal diversity between the different media used (Figure 4).

**Figure 5:**
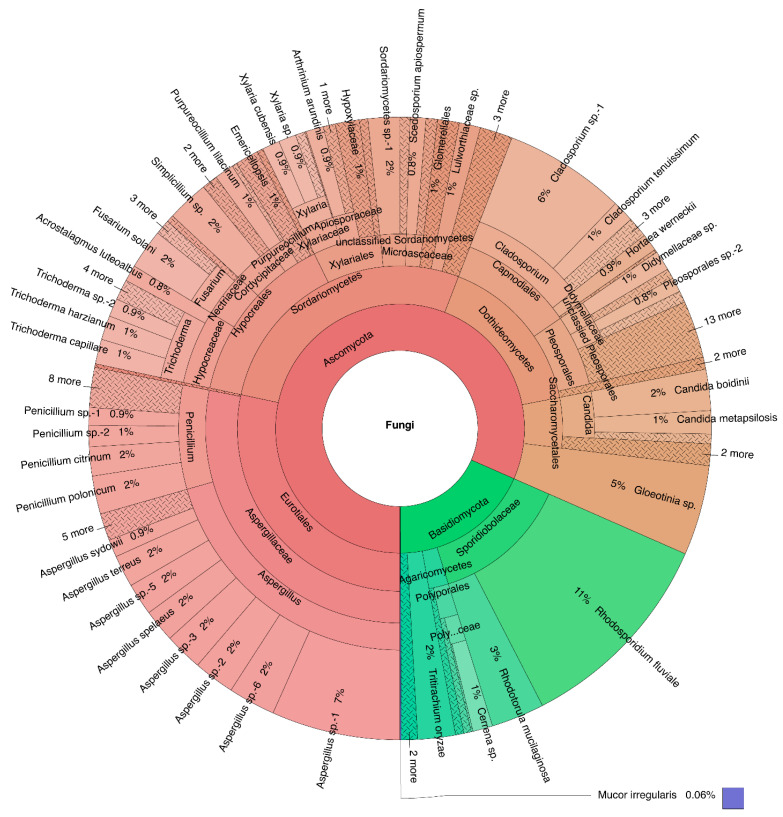
Krona chart showing the taxonomic classification of fungi isolated from *Pterocladiella capillacea* based on their relative abundance. The outer circle represents the isolated species and the inner circles represent their higher taxonomic classification. An interactive version of this chart is provided as Figure S2.

Figure 5 shows the taxonomic classification of fungi cultured from *P. capillacea*. Ascomycota was represented by 119 species in 57 genera (2600 isolates, 81.58%), Basidiomycota by 9 species in 9 genera (585 isolates, 18.36%), and Mucoromycota by 1 unidentified species (2 isolates, 0.06%). For Ascomycota, the most abundant class was Eurotiomycetes (903 isolates, 28.33%), followed by Sordariomycetes (877 isolates, 27.52%) and Dothideomycetes (513 isolates, 16.10%). The five dominant fungal orders were Eurotiales (903 isolates, 28.33%), Hypocreales (459 isolates, 14.40%), Capnodiales (305 isolates, 9.57%), Pleosporales (188 isolates, 5.90%), and Xylariales (179 isolates, 5.62%). The percentage occurrence of other fungal orders was less than 5%. The most abundant fungal genus was the *Aspergillus*, with a total 656 isolates that accounted for 20.58% of the overall occurrence, followed by *Cladosporium* (271 isolates, 8.50%) and *Penicillium* (241 isolates, 7.56%). Species with the highest overall occurrence were *Aspergillus* sp. 1 (213 isolates, 6.68%), *Cladosporium* sp. 1 (201 isolates, 6.31%), and *Gloeotinia* sp. (149 isolates, 4.68%). In terms of richness, the most speciose genera on *P. capillacea* were *Aspergillus* (13 species), *Penicillium* (12 species), *Trichoderma* (7 species), and *Cladosporium* and *Xylaria* (5 species).

For Basidiomycota, Microbotryomycetes and Sporidiobolales (both 13.62%, 434 isolates) were the dominant class and order, respectively. *Rhodosporidium* (*R. fluviale*) and *Rhodotorula* (*R. mucilaginosa*) accounted for 10.86% (346 isolates) and 2.76% (88 isolates), respectively.

### 3.2. Fungal Diversity in Healthy and Dead Thalli

The isolation frequencies for healthy and dead thalli were 56.37% and 61.23%, respectively (Figure 2). A significant difference in fungal diversity between the healthy and dead thalli was observed (Figure 4, Table 1).

**Figure 6:**
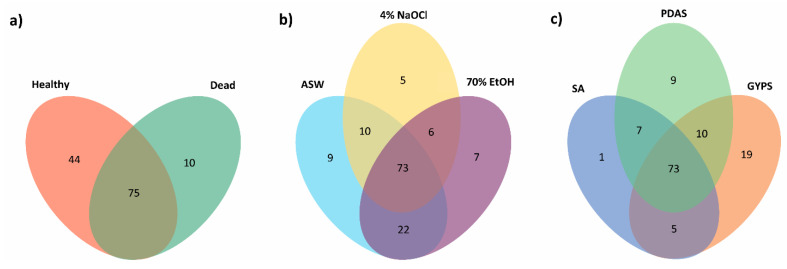
Venn diagrams showing fungal species richness (**a**) in healthy and dead thalli, (**b**) in thalli under different sterilization regimes (artificial seawater (ASW), 70% ethanol (70% EtOH), and 4% sodium hypochlorite (4% NaOCl)), and (**c**) in thalli isolated on different media (seawater agar (SA), glucose-yeast extract-peptone seawater agar (GYPS), and potato dextrose seawater agar (PDAS)).

More fungal isolates were cultured from the healthy thalli (2281 isolates, 71.57% occurrence) than the dead thalli (906 isolates, 28.43%) (Table S1). A total of 119 fungal species were obtained from healthy thalli and 85 species from dead thalli (Figure 6a). Additionally, 44 and 10 species were exclusively isolated from healthy and dead thalli, respectively, with 75 common species (Figure 6a).

**Figure 7:**
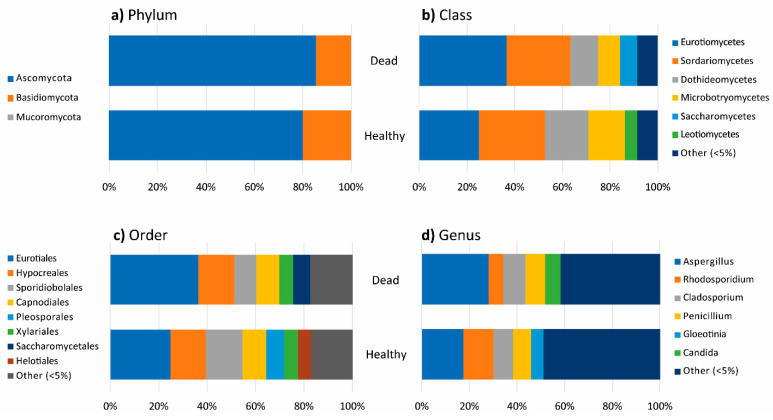
Bar chart showing diversity of fungi at (**a**) phylum, (**b**) class, (**c**) order, and (**d**) genus levels in healthy and dead thalli.

The taxonomic classification of fungi isolated from the healthy and dead thalli of *P. capillacea* was similar at the phylum, class, and order levels, but with a different percentage occurrence. A slightly higher occurrence of Ascomycota was found in dead than healthy thalli and the reverse was true for Basidiomycota (Figure 7a).

Percentage occurrences of Eurotiomycetes and Saccharomycetes were higher in dead thalli, while those of Dothideomycetes and Microbotryomycetes were higher in healthy thalli (Figure 7b). Fungi isolated from the healthy thalli belonged to 23 different fungal orders and the dominant genera were *Aspergillus* (17.58%), *Rhodosporidium* (12.71%), and *Cladosporium* (8.15%). Fungi isolated from the dead thalli belonged to 20 fungal orders and the dominant fungi were *Aspergillus* spp. (28.15%)*, Cladosporium* spp. (9.38%), and *Penicillium* spp. (7.95%) (Figure 7c,d).

Lulworthiaceae sp. (1.53%) and Didymellaceae sp. (1.45%) only occurred in the healthy thalli. From the 10 taxa only isolated from the dead thalli, *Bisifusarium* sp. (0.44%), *Gliomastix* sp. (0.22%), and *Westerdykella* sp. (0.11%) had the highest percentage occurrence (Table S1).

### 3.3. Effect of Algal Surface Washing/Sterilization Methods on Fungal Diversity

The isolation frequencies from the different surface washing/sterilization methods were the highest in seawater (88.38%), followed by 70% ethanol (54.74%) and 4% sodium hypochlorite (30.33%) (Figure 2). Thalli washed with seawater (111 species) had the highest species richness, followed by 70% ethanol (105 species) and 4% sodium hypochlorite (91 species) (Figure 6b).

**Figure 8:**
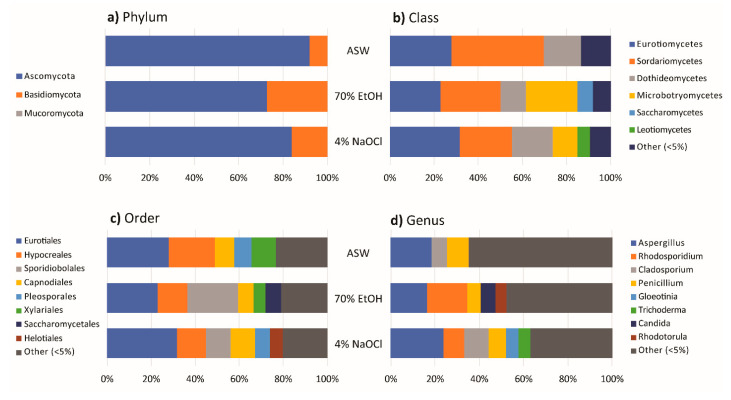
Bar chart showing diversity of fungi at the (**a**) phylum, (**b**) class, (**c**) order, and (**d**) genus levels of thalli sterilized with artificial seawater (ASW), 70% ethanol (70% EtOH), and 4% sodium hypochlorite (4% NaOCl).

Seventy-three species could be isolated from all sterilization methods. Nine, five, and seven species were only cultured from one washing/sterilization method, being seawater, sodium hypochlorite and ethanol, respectively (Figure 6b). Fungi of the Venturiales were only isolated from the ethanol-sterilized samples; Mucorales were not isolated from the sodium hypochlorite-sterilized thalli (Table S1). At phylum level, a higher occurrence of Basidiomycota was observed on thalli sterilized by 70% ethanol (Figure 8a).

At the class level, a much higher occurrence of Sordariomycetes (mainly in Hypocreales) was isolated from thalli sterilized with sodium hypochlorite, while Microbotryomycetes’ occurrence (mainly in Sporidiobolales) was the highest in thalli sterilized with ethanol (Figure 8b,c). The dominant genera in seawater-washed thalli were *Aspergillus* (23.68%), *Cladosporium* (11.08%), *Rhodosporidium* (9.38%), and *Penicillium* (7.74%). *Rhodosporidium* (17.92%), *Aspergillus* (16.42%), *Candida* (6.61%), and *Penicillium* (6.21%) were the dominant genera in the ethanol-sterilized samples. *Aspergillus* (18.26%) and *Penicillium* (9.75%) and *Cladosporium* were the dominant genera in hypochlorite-sterilized thalli (7.05%) (Figure 8d).

### 3.4. Effect of Culture Media on Fungal Diversity

**Figure 9:**
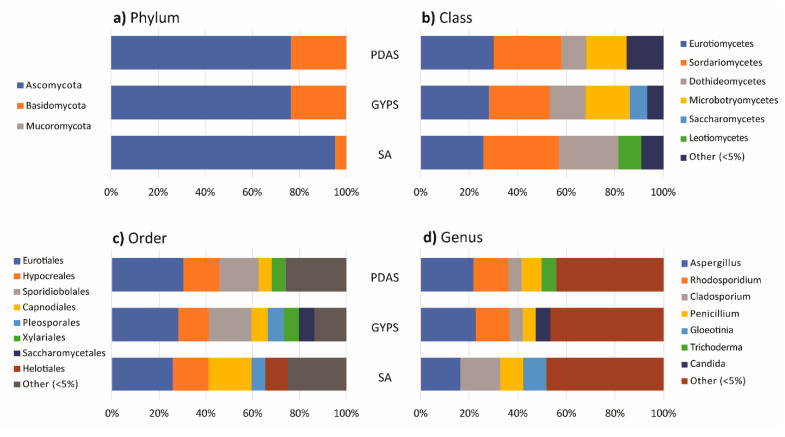
Bar chart showing diversity of fungi at the (**a**) phylum, (**b**) class, (**c**) order, and (**d**) genus levels isolated on seawater agar (SA), glucose-yeast extract-peptone seawater agar (GYPS), and potato dextrose seawater agar (PDAS).

The isolation frequencies on SA, GYPS, and PDAS were 50.13%, 62.10%, and 60.47%, respectively (Figure 2). The highest number of isolates and fungal species were recovered from GYPS with 1211 isolates and 112 species, followed by PDAS (1091 isolates, 104 species) and SA (885 isolates, 86 species) (Figure 6c). Further, 1, 9, and 19 species were only isolated from SA, PDAS, and GYPS, respectively (Figure 6c). On SA, a lower occurrence of Basidiomycota (mainly in Sporidiobolales, Microbotryomycetes) (Figure 9a,b and Table S1), but a higher occurrence of Capnodiales and Helotiales was observed (Figure 9c).

The most represented orders were Eurotiales (25.99%), Capnodiales (18.31%), Hypocreales (15.14%), and Helotiales (9.49%), and the dominant fungal genera were *Cladosporium* (16.61%), *Aspergillus* (16.38%), and *Gloeotinia* (9.49%) (Figure 9d). A comparatively high occurrence of Saccharomycetes (i.e., Saccharomycetales) was isolated from GYPS (Figure 9b,c). Additionally, 21 orders were isolated from GYPS with Eurotiales (28.24%), Sporidiales (18.33%), and Hypocreales (12.96%) being the dominant orders (Figure 9c), while the dominant genera were *Aspergillus* (22.71%), *Rhodosporidium* (13.71%), *Candida* (5.78%), *Cladosporium,* and *Penicillium* (5.53%) (Figure 9d). Venturiales and Mucorales were only isolated from GYPS. *Aspergillus* (21.63%), *Rhodosporidium* (14.67%), and *Penicillium* (8.34%) were the dominant genera on PDAS with Eurotiales (30.34%), Sporidiales (16.77%), and Hypocreales (15.40%) being the most dominant orders (Figure 9c,d).

Finally, 73 fungal species were common for all media, including *Aspergillus* spp. (20.58%), *Rhodosporidium* sp. (10.86%), and *Cladosporium* (8.50%) with the highest percentage occurrence (Table S1).

## 4. Discussion

### 4.1. Fungal Community

This is the first report of culturable fungi associated with *Pterocladiella capillacea*, and the results revealed a high diversity of culturable fungi with 129 species in 67 genera. The Ascomycota was dominant in *P. capillacea*, in which Eurotiales, Hypocreales, Pleosporales, and Capnodiales were the most abundant. Fungi of these orders were shown to be halotolerant and halophilic species [48], which may explain their close association with the alga. Xylariales are predominantly terrestrial, while many occur as endophytes of plants, and they produce secondary metabolites against bacterial and fungal pathogens [49]. In the marine environment, the xylarialean fungi also occur as endophytes of seagrasses, mangrove plants, and macroalgae [50]. The xylarialean fungi (as well as other taxa) isolated from *P. capillacea* in this study might also produce antimicrobial substances to protect the alga from diseases. A *Xylaria* species isolated from the red alga *Bostrychia tenella* produced cytochalasin D, an antibiotic compound, which may confirm this conclusion [51]. Nevertheless, *Chondrostereum* sp. NTOU4196 and *Phoma* sp. NTOU4195 isolated in this study produced novel chemical structures in spent culture liquid with anti-inflammatory properties, suggesting a prolific source of bioactive compounds in the marine environment [52,53].

*Aspergillus, Penicillium* (Eurotiales)*, Cladosporium* (Capnodiales), and *Trichoderma* (Hypocreales) are cosmopolitan in distribution and can be found in terrestrial and aquatic environments. The widespread occurrence of these taxa in the marine environment may suggest their passive migration from terrestrial habitats [54]. Their high occurrence on *P. capillacea* is not surprising as numerous species of these genera can be found in the marine environment [55]. Some studies suggested that these common genera of fungi were able to tolerate or degrade antifungal compounds in macroalgae, which could explain their wide host colonization [19]. However, it is important to point out that the culture-dependent technique used in this study may bias toward the isolation of these fast-growing taxa [56]. The culture-independent approach revealed a different diversity of fungi associated with the brown alga *Fucus serratus* from the isolation technique [25].

Species of *Aspergillus* and *Penicillium* were among the most common taxa in the marine environment [55,57,58]; they are common endosymbionts of different seaweeds [12,22,59,60] and seagrasses [61]. *Aspergillus* spp., apart from dominating the endosymbiont assemblage of seaweeds [23], also dominated the fungal assemblages of marine invertebrates [32,62,63]. *Aspergillus* spp. are known to be halotolerant and have the genetic capacity to adapt to salinity pressure and this plasticity may confer an ecological advantage over others [48,64]. *Penicillium* is one of the most common genera isolated from macroalgae [13]. *Penicillium* spp. isolated from red algae in maritime Antarctica displayed carrageenolytic and agarolytic activities, suggesting that they play a role in recycling carbon when the algae die [65]. Both *Aspergillus* and *Penicillium* species are known to produce antimicrobial secondary metabolites [32,60] and, thus, may protect macroalgae from microbial infection. In a study of the mycobiota of the red *alga Asparagopsis taxiformis*, two out of the five species isolated belonged to *Cladosporium* [66].

Yeast species *Rhodosporidium fluviale* and *Rhodotorula mucilaginosa* dominated the occurrence of Basidiomycota in *P. capillacea*. Basidiomycetous yeasts are common in the marine environment [67]. Few filamentous Basidiomycota can be found in the marine environment, possibly because they are salinity sensitive [68]. The known terrestrial Agaricomycetes isolated from *P. capillacea* might only represent a superficial association with the alga [69]. Mucoromycota is uncommon in the marine environment [55] and so it seems reasonable that only two isolates were cultured from *P. capillacea*. Whether isolation of marine Mucoromycota requires special techniques or media needs further study.

The isolation frequencies for healthy and dead thalli were similar but a higher percentage occurrence and a higher species richness were observed in healthy thalli. The fungal community between healthy and dead thalli of *P. capillacea* was highly similar (75 taxa), while the percentage occurrences of unique species were low (<1%), especially in dead thalli. However, Lulworthiaceae sp. and Didymellaceae sp. (>1%) may play a role in healthy thalli.

*Pterocladiella capillacea* is a perennial red alga that can be found at the sampling site in Keelung, Taiwan. No disease symptom was observed during sample collection, suggesting the fungi cultured in this study do not cause diseases of the alga. However, *Aspergillus ochraceus*, *A. terreus,* and a *Phoma* species induced bleaching disease symptoms in healthy, non-axenic cultures of *Kappaphycus alvarezii* [18]. Many cultured taxa in this study, as described above, have been proven to produce antimicrobial compounds. *Pterocladiella capillacea* might have formed a symbiotic relationship with some of its associated fungi by exchanging nutrients from photosynthesis for protection from microbial diseases.

### 4.2. Isolation Methods

In this study, sodium hypochlorite (4%) was found to be an effective surface sterilizer based on its low isolation frequency when compared with 70% ethanol. Without surface sterilization, the isolation frequency was three times higher (washed with sterilized seawater). The Kruskal–Wallis test also suggested a significant effect of algal surface washing/sterilization methods on fungal diversity, and consequently, species richness was highest in seawater, followed by ethanol and sodium hypochlorite, but the dominant species were similar for the three methods of surface washing/sterilization. The percentage occurrence of unique species from each of the three methods of surface washing/sterilization was very low. Mucorales might only associate with *P. capillacea* epiphytically, as this group was not found in sodium hypochlorite-sterilized samples. To study total culturable diversity of fungi associated with *P. capillacea*, washing of algal thalli with sterile seawater is recommended, otherwise sodium hypochlorite can be used to sterilize the algal surface to study the endophytic assemblage of fungi.

The SA, PDAS, and GYPS used for the isolation in this study are common media to study marine fungi, especially those associated with macroalgae [15,18,19,21,22,32,33,34,35]. For media, high isolation frequencies on PDAS and GYPS were reasonable, as glucose is the major carbon in these media, which favors growth of saprobic species over endophytic species. The yeasts *Rhodosporidium fluviale*, *Rhodotorula mucilaginosa* (Microbotryomycetes, Basidiomycota), and *Candida* spp. (Saccharomycetes, Ascomycota) generally grow well in media rich in glucose [70], and these taxa had high occurrence in PDAS and GYPS. The comparatively lower isolation frequency on SA can be explained by the minimal nutrients it contains [32]. The effect of natural against artificial seawater and ‘aged’ against ‘newly collected’ natural seawater is unknown, but decomposition of organic matter in natural seawater during the ‘aging’ process may enrich the seawater with more readily available nutrients [71]. However, species richness on the three media was similar and only differed in overall occurrence, and the Kruskal–Wallis test also suggested no significant effect of media on fungal diversity. The use of both GYPS and PDAS as the isolation media was sufficient to study the culturable diversity of fungi associated with *P. capillacea* and possibly other macroalgae.

## 5. Conclusions

A high diversity of epiphytic and endophytic fungi was associated with the red alga *Pterocladiella capillacea,* with 129 species in 67 genera. Filamentous Ascomycota (Eurotiales, Capnodiales, Hypocreales, Pleosporales) dominated the community with many basidiomycetous yeasts (Sporidiobolales) and few Mucoromycota. Many cultured fungi (*Aspergillus*, *Penicillium*, Xylariales) are known producers of antimicrobial secondary metabolites and may symbiotically associate with the alga. Sodium hypochlorite is an effective surface sterilizer to study the endophytic mycobiota of macroalgae. Media used for fungal isolation did not have a significant effect on species richness, but only an effect on the overall occurrence.

## Figures and Tables

**Table 1:** Diversity indices of fungal communities isolated from healthy/dead thalli of *Pterocladiella capillacea* after surface washing/sterilization by artificial seawater (ASW), 70% ethanol (70% EtOH), and 4% sodium hypochlorite (4% NaOCl) plated on seawater agar (SA), glucose-yeast extract-peptone seawater agar (GYPS), and potato dextrose seawater agar (PDAS).

.	Thalli		Surface Washing/Sterilization			Medium			Total
	Healthy	Dead	ASW	70% EtOH	4% NaOCl	SA	GYPS	PDAS	
Total No. of isolates (Total Abundance), N	2281	906	1706	999	482	885	1211	1091	3187
Richness (Total number of Taxa in the community), S	119	85	111	105	91	86	112	104	129
Species Richness (Margalef): *d*	15.2605	12.3365	14.7812	15.0577	14.5680	12.5265	15.6356	14.7251	15.8674
Shannon-Wiener Diversity Index: *H*′	4.0335	3.8510	3.9623	3.8180	4.0923	3.7189	4.0107	3.8901	4.0982
Pielou’s Evenness: *J*′	0.8440	0.8668	0.8413	0.8204	0.9072	0.8349	0.8500	0.8376	0.8433
Simpson’s Dominance Index: *D*	0.0349	0.0312	0.0342	0.0489	0.0233	0.0450	0.0379	0.0408	0.0310

## Data Availability

All sequence data associated with this study have been submitted to the NCBI.

## Supplementary Materials

The following are available online at https://www.mdpi.com/article/10.3390/jof7080651/s1, Figure S1: Schematic diagram of experimental, Figure S2: An interactive version of Krona chart showing the taxonomic classification of fungi isolated from *Pterocladiella capillacea* based on their relative abundance, Table S1: Taxonomic information and overall fungal isolates.
