# Supplementary figures and images for: Culturable Fungal Community of Pterocladiella capillacea in Keelung, Taiwan: Effects of Surface Sterilization Method and Isolation Medium
Source: J Fungi (Basel). 2021 Aug 11;7(8):651. doi: 10.3390/jof7080651 (PMC8399158; doi:10.3390/jof7080651)

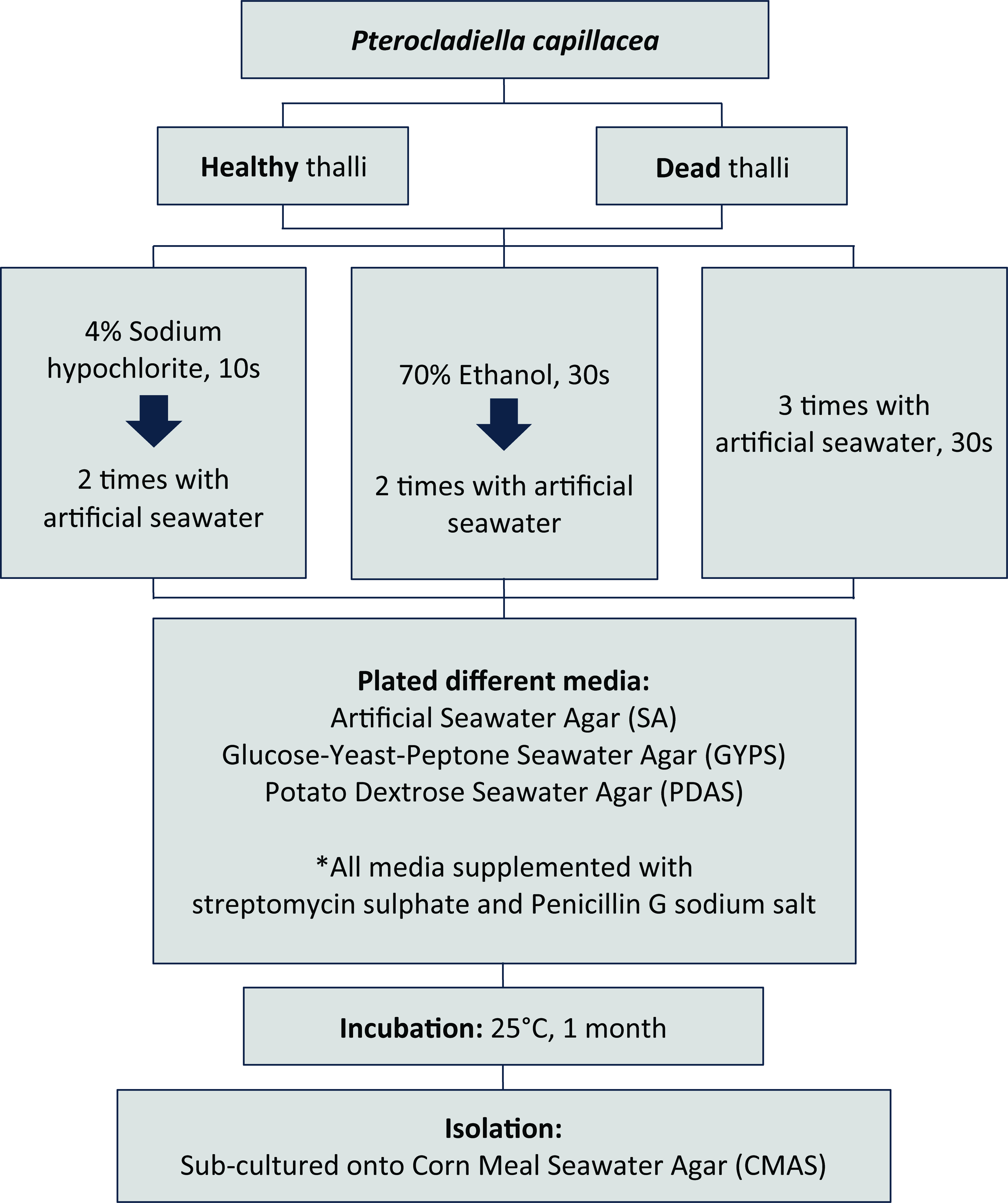

Supplement: Supplementary file 1 [file jof-07-00651-s001.zip › Revised_Supplementary_JoF/Fig S1. Schematic diagram of experimental.tif]
